# Folic Acid and Taurine Alleviate the Impairment of Redox Status, Immunity, Rumen Microbial Composition and Fermentation of Lambs under Heat Stress

**DOI:** 10.3390/ani14070998

**Published:** 2024-03-25

**Authors:** Bibo Li, Ke Wu, Guoqing Duan, Weiqi Yin, Mingkai Lei, Yining Yan, Youshe Ren, Chunxiang Zhang

**Affiliations:** College of Animal Science, Shanxi Agricultural University, Taiyuan 030031, China; libibo1988@126.com (B.L.); wuke1227@163.com (K.W.); gqduan0423@163.com (G.D.); yin18434762771@163.com (W.Y.); electorboy@163.com (M.L.); 15934508623@163.com (Y.Y.)

**Keywords:** sheep, heat stress, folic acid and taurine, physiological indices, oxidant resistance, immunomodulation, rumen fermentation and microbiota

## Abstract

**Simple Summary:**

Heat stress (HS) constantly happens to dry lot feeding sheep during the summer, inevitably causing substantial losses to animal production due to its unfavorable effects on health, welfare and production performance. In the current study, HS played disadvantageous roles in growth performance, physiological indices, antioxidative capacity, immunomodulation, ruminal fermentation and microbial composition. Dietary supplementation with folic acid and taurine can effectively ameliorate adverse effects of HS by improving the proportion of beneficial bacteria such as *Quinella* and *Succinivibrio* and decreasing the abundance of harmful bacteria such as *Asteroleplasma*. This study provides a practical approach for the application of folic acid and taurine in HS-challenged sheep.

**Abstract:**

The aim of this study was to investigate if the supplementation of folic acid and taurine can relieve the adverse effects of different levels of heat stress (HS) on growth performance, physiological indices, antioxidative capacity, immunity, rumen fermentation and microbiota. A total of 24 Dorper × Hu crossbred lambs (27.51 ± 0.96 kg) were divided into four groups: control group (C, 25 °C), moderate HS group (MHS, 35 °C), severe HS group (SHS, 40 °C), and the treatment group, under severe HS (RHS, 40 °C, 4 and 40 mg/kg BW/d coated folic acid and taurine, respectively). Results showed that, compared with Group C, HS significantly decreased the ADG of lambs (*p* < 0.05), and the ADG in the RHS group was markedly higher than in the MHS and SHS group (*p* < 0.05). HS had significant detrimental effects on physiological indices, antioxidative indices and immune status on the 4th day (*p* < 0.05). The physiological indices, such as RR and ST, increased significantly (*p* < 0.05) with the HS level and were significantly decreased in the RHS group, compared to the SHS group (*p* < 0.05). HS induced the significant increase of MDA, TNF-α, and IL-β, and the decrease of T-AOC, SOD, GPx, IL-10, IL-13, IgA, IgG, and IgM (*p* < 0.05). However, there was a significant improvement in these indices after the supplementation of folic acid and taurine under HS. Moreover, there were a significant increase in *Quinella* and *Succinivibrio*, and an evident decrease of the genera *Rikenellaceae_RC9_gut_group* and *Asteroleplasma* under HS (*p* < 0.05). The LEfSe analysis showed that the genera *Butyrivibrio*, *Eubacterium_ventriosum_group*, and f_Bifidobacteriaceae were enriched in the MHS, SHS and RHS groups, respectively. Correlated analysis indicated that the genus *Rikenellaceae_RC9_gut_group* was positively associated with MDA, while it was negatively involved in IL-10, IgA, IgM, and SOD (*p* < 0.05); The genus *Anaeroplasma* was positively associated with the propionate and valerate, while the genus *Succinivibrio* was negatively involved in TNF-α (*p* < 0.05). In conclusion, folic acid and taurine may alleviate the adverse effects of HS on antioxidant capacity, immunomodulation, and rumen fermentation of lambs by inducing changes in the microbiome that improve animal growth performance.

## 1. Introduction

Heat stress (HS) has become one of the greatest challenges for the livestock industry worldwide as global warming intensifies. It is reported that environmental temperature has increased by 1.0 °C for over 200 years and has the potential to continue to rise by another 1.5 °C between 2030 and 2052 [1]. It is generally accepted that the augmented magnitude of global temperature, more frequent heat waves due to global warming, and the migration of animal production to tropical regions to satisfy the increased demand of animal-edible animal products, have together exacerbated the occurrences of HS in livestock [2]. HS occurs when an animal is continuously exposed to elevated ambient temperature and humidity, which compromises the animal’s ability to dissipate sufficient heat, whether metabolically produced or absorbed, to maintain the normal core body temperature [3,4,5]. Sheep, as a case of ruminants, have intensive metabolic heat production due to feed fermentation in the rumen and a relatively small surface: volume ratio, and are particularly susceptible to HS due to a limited capacity for heat dissipation [5,6]. As one of the major environmental stressors, HS not only induces a range of negative effects comprising deprived appetite, decreased feed intake, and reduced weight gain, but is deleterious to reproduction performance, immune response, antioxidative activity, gastrointestinal microbiota and relevant function [4,7,8,9]. Hence, HS is often defined as a sensation of discomfort that evokes aberrant physiological reactions, i.e., increased body temperature, accelerated respiratory rate, and elevated heart rate [8]. Nevertheless, the deleterious consequences of HS for animal health and production are likely to continue in the future, especially if the production traits are preferentially selected for over traits for thermos tolerance and climate adaptation.

Consequently, it is particularly imperative to develop practical strategies that can alleviate the detrimental effects of HS on animals. Although the regulation of ambient temperature and relative humidity to mitigate HS is the most directly effective approach, the high cost of the control system greatly restricts its application to farm animals [10]. However, the modification of the diet with functional additives, such as the supplement of functional additives (including minerals), vitamins, active ingredients of Chinese traditional herbs, amino acids, probiotics, etc., has become an economically feasible mitigation technology to reduce the negative consequences induced by HS [9,11,12]. Folic acid, a B vitamin, is vital to embryonic formation and fetal development because it plays crucial roles in the repair and methylation of DNA, gene expression and modification, and amino acid metabolism [13]. Moreover, folic acid exhibits an evident antioxidant activity due to its coenzyme (tetrahydrofolate), including purine and pyrazine type rings. And the hydroxyl group located on the purine ring can react with active free radical, which makes folic acid play an important role in inhibiting oxidative stress [14,15]. It was reported that the supplement of folic acid at 1.5 mg/kg significantly improved the broilers’ total antioxidant capacity (T-AOC), catalase enzyme activity (CAT), and superoxide dismutase (SOD) [14]. Also, a folic acid supplement in diet was beneficial for maternal or offsprings’ immune status in a sheep model [16]. Another study showed that dietary-coated folic acid improved rumen fermentation, nutrient digestibility, and growth performance in bulls [17].

Taurine, a non-standard amino acid, is often used as an effective feed additive to mitigate the physiological consequences of stress in animals during adverse conditions, such as HS, intensive feeding, oxidative stress, and endotoxins [18]. Taurine has been implicated in alleviating the biological consequences of stress in animals due to its effects as an anti-oxidant, anti-inflammatory immunomodulator, and its role in restoring membrane integrity, maintaining cellular homeostasis, and ensuring energy metabolism balance, osmoregulation, and neuroprotection [19,20,21]. Studies of broilers, supplemented at 5 g/kg taurine of basal diet reared under HS (32 °C, 55 ± 5% RH for 14 d), indicated that taurine had no significant effects on the growth performance of HS broilers but decreased reactive oxygen species (ROS) and malonaldehyde (MDA) and attenuated mitochondrial damage [22,23]. Apart from eliminating ROS directly, taurine can also take crucial roles in preserving or enhancing the activities of the antioxidant enzymes, including SOD, GPx, and CAT, and protecting oxidative damage from HS [24]. Additionally, taurine supplementation in the diet can also improve the carcass quality of broilers exposed to chronic HS via promoting protein synthesis and suppressing protein degradation of breast muscles, although without producing significant effects on growth performance damage [25]. In brief, studies regarding the effects of folic acid or taurine on livestock under HS have mostly focused on poultry or rats; however, little information was obtained on the ruminants, especially on sheep or goats.

Based on the beneficial effects of folic acid and taurine, we hypothesized that they can ameliorate damage to immunity and antioxidant capacity induced by chronic HS and modulate the microbial community in rumen. Hence, the object of this study was to investigate the protective effects of folic acid and taurine on the physiological indices, oxidative stability, immune status, and ruminal fermentation of Dorper–Hu crossbred male lambs and to probe its molecular mechanism, to provide new insights for mitigating HS. 

## 2. Materials and Methods

### 2.1. Experimental Design and Sampling

All the experimental procedures with sheep used in the present study had been given prior approval by the Experimental Animal Manage Committee of Shanxi Agricultural University. A total of 24 6-month-old crossbred male lambs (Dorper × Hu) with an average weight of (27.51 ± 0.96) kg were used in the study. All lambs were selected following thorough examination for general health and were vaccinated and dewormed according to standard practices on the farm. The climate chamber was sanitized prior to the test. Based on a single factor and completely random experiment design, all lambs were allocated to four groups: the control group (C, 25 °C), the moderate HS group (MHS, 35 °C), the severe HS group (SHS, 40 °C), the coated folic acid and taurine addition group, or the relief group under severe HS (RHS, 40 °C, 4 and 40 mg/kg BW/d coated folic acid and taurine, respectively); each group was in an independent climate chamber, with six replicates in each group (one sheep in a pen). The RH was maintained at 50% throughout the test period. The lambs were acclimatized to the climate chamber (25 °C, 50% RH) for 5 d in group pens and then housed in individual pens for 5 d before the heat treatment. The experimental period was for 7 d. In the C group, the ambient temperature was maintained at 25 °C every day, while in the MHS and SHS groups, it started to rise from 25 °C at 8:00 and reached to 35 °C and 40 °C at 14:00, respectively; after duration for 2 h, the temperature began to decrease and drop to 25 °C at 20:00. The temperature variation in the RHS group was identical to the SHS group. Lambs were individually fed a complete formula granulated feed, formulated as per NRC (2007). The ingredients and nutrients of the experiment diet are provided in Table S1. Lambs were fed twice daily (7:00 and 18:00) and had free access to clean water. At the beginning and end of the experiment, each lamb was weighted before morning feeding to calculate the average daily gain (ADG). The amount of offered feed and corresponding residues provided every day were recorded to determine the average daily feed intake (ADFI). Temperature humidity index (THI) was adopted to measure the severity of HS. THI was calculated as follows: THI = td − (0.55 − 0.55 × RH) (td-58), while td = °C × 1.8 + 32.

Blood samples (10 mL per lamb) were collected from the jugular vein at 16:00 h on the 1st, 4th, and 7th day of the experiment. Immediately, blood samples were placed in a water bath at 37 °C for 30 min and centrifuged at 3000× *g* for 10 min to harvest serum, before being aliquoted to microcentrifuge tubes and frozen at −20 °C for serum parameter analysis. In addition, at 16:00 h on Day 4, an approximate volume of 25 mL rumen digesta per lamb was collected via vacuum pump with an oral stomach tube. A part of the ruminal digesta (10 mL) was immediately snap-frozen in liquid nitrogen and then stored in a −80 °C freezer for microbial DNA extraction, while another part was filtered with 4 layers of gauze to obtain ruminal fluid for pH determination. Subsequently, 25% (*w*/*v*) metaphosphoric acid was added in rumen liquid and preserved at −20 °C for later analysis of rumen ferment parameters.

### 2.2. Measurements of Physiological Indices, Inflammatory Cytokine, and Antioxidative Capacity in 1st Crossbred (Dorper × Hu) Male Lambs

Physiological parameters, respiration rate (RR), heat rate (HR), skin temperature (ST) and rectal temperature (RT) were measured at 9:00, 13:00, 15:00 and 17:00 on Day 3, 4 and 5 of the formal experiment. The RR was determined by counting the flank movements for 30 s and taking the average value from 3 consecutive measurements. HR was reflected by the pulse rate, which was measured by palpating the pulse beat in the left hoof for 60 s and then expressed in beats per minute. An infrared thermometer gun was used to measure ST obtained via determining the average of the temperature of the forequarters, midquarters and hindquarters of the lamb. RT was determined by placing an electronic thermometer into the rectum for 3 min until a stable temperature was shown on the screen [3].

The serum antioxidant indices for malondialdehyde (MDA), total antioxidant capacity (T-AOC), glutathione peroxidase (GPx), and superoxide dismutase (SOD) were measured using commercial kits (Jian cheng biotechnology, Nanjing, China) [8]. The concentration of TNF-α, IL-1β, IL-10, IL-13, IgA, IgG, IgM, and HSP70 was measured using commercial ELISA kits (Mei lian biotechnology, Shanghai, China) [4]. The ELISA was conducted on a RT6100 microplate reader (Rayto life and analytical sciences company, Shenzhen, China).

### 2.3. Rumen Fermentation

The concentration of volatile fatty acid (VFA), which consisted of acetate, propionate, isobutyrate, butyrate, isovalerate, and valerate, was measured using gas chromatography (GC) equipped with a 30 m × 0.53 mm × 1.00 μm capillary column (Aglient 7890B, Santa Clara, CA, USA) [26]. Colorimetric method of sodium nitroferricyanide-sodium hypochlorite was selected to determine the content of NH_3_-N with a UV spectrophotometer (UV1000, Shanghai, China).

### 2.4. Rumen Bacterial DNA Extraction, Amplification, Sequencing and Analysis

Approximately 300 mg of homogenized ruminal digesta sample was isolated to extract microbial DNA using a bead beating method, followed by sodium dodecyl sulfate/guanidine thiocyanate (SDS/GITC) and phenol-chloroform isoamyl extraction [27]. The quality and concentration of DNA was determined on a Nanodrop spectrophotometer. The qualified DNA with more than 1.80 of OD260/280 was used for further PCR amplification. The primers 338F and 806R were used to amplify the V3-V4 hypervariable region of the bacterial 16S rRNA gene in a 20 μL PCR reaction mixture [28]. The PCR product was extracted from 2% agarose gel and purified using a PCR Clean-Up Kit (YuHua, Shanghai, China). After this, purified amplicons were pooled in equimolar amounts and paired-end sequenced on an Illumina PE300 platform (Illumina, San Diego, CA, USA) according to the standard protocols by Majorbio Bio-Pharm Technology Co. Ltd. (Shanghai, China). Raw FASTQ sequences were demultiplexed, quality filtered with fastp (0.19.6) [29] and merged with FLASH (v.1.2.11) [30]. Then, the resulting sequences were denoised as amplicon sequence variants (ASVs) using DADA2 plugin in QIIME2 (v.2020.3) [31]. Taxonomic assignment of ASVs was performed to the genus level and aligned to the SILVA 16S rRNA database (v138) using the Naïve bayes consensus taxonomy classifier.

Bioinformatic analysis of the ruminal microbiota was conducted using the Majorbio Cloud platform “https://cloud.majorbio.com (accessed on 1 May 2022)”. Rarefaction curves were used to estimate the sequencing depth. Based on ASVs, the α diversity was performed to assess the bacterial richness and diversity. The similarity of microbial community among different groups was visualized by principal coordinate analysis (PCoA),based on Bray–Curtis distance and analysis of similarity (ANOSIM) using Vegan v3-3.1 package. The linear discriminant analysis (LDA) effect size (LEfSe) was performed to identify the significantly abundant taxa (phylum to genus) of bacteria among each group (LDA > 3, *p* < 0.05).

### 2.5. Statistical Analysis

The significant difference of growth performance, physiological indices, ruminal fermentation parameters, α diversity (Chao, Shannon), and the relative abundant of predominant bacterial taxa, were analyzed using one-way ANOVA with Ducan’s post hoc comparison in SPSS 24.0. The comparison of antioxidative indices and inflammatory cytokine was performed in SPSS 24.0 by using a repeated measurement analysis within General Linear Mode (GLM). Correlation analysis between the environmental factors and microbiota were calculated by Sperman’s correlation test using the R “heatmap” package. The value of *p* < 0.05 was statistically significant.

## 3. Results

### 3.1. THI and Growth Performance

Figure S1 illustrates the daily THI in each group. It shows that the average value of THI between 14:00 and 16:00 in C, MHS, SHS, and RHS were 71.54, 84.30, 88.48, and 89.91, respectively. The obtained values indicate the following: values <72 = absence of HS; 72 to <79 = mild HS; 79 to <88 = moderate HS; and over 88 = severe HS. These data mirrored that the lambs in each chamber were in a corresponding HS status. Moreover, compared to the C group, HS significantly decreased the ADG of lambs (*p* < 0.05), but there was no significant difference between the MHS and SHS groups. The ADG of lambs in the RHS group was markedly higher than in the MHS and SHS groups (*p* < 0.05), which suggested that the supplement of folic acid and taurine can effectively relieve the deleterious influence of HS on ADG. Although there was no significant difference between each group, HS reduced the ADFI of lambs to a certain extent. And the supplement of folic acid and taurine can improve the ADFI under HS.

### 3.2. Effects of Folic Acid and Taurine on the HR, RR, ST and RT of Du-Hu Crossed Ram under HS

**Figure 1:**
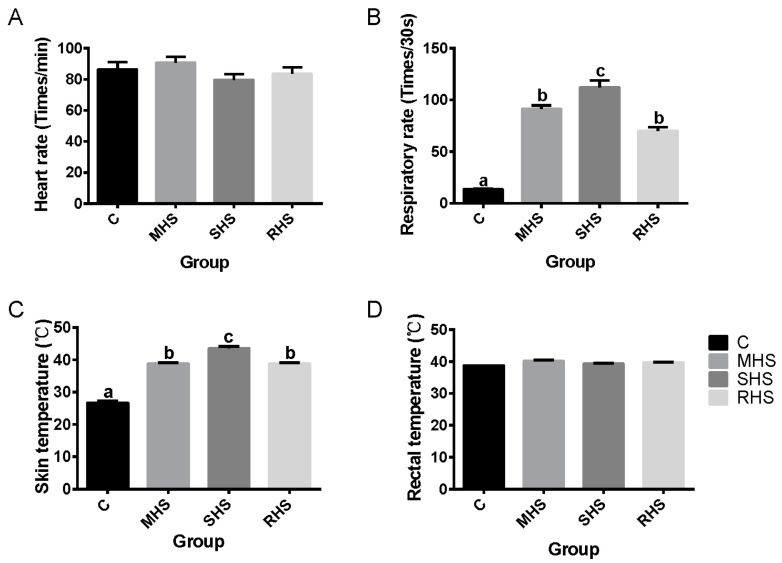
Comparison of heart rate (HR) (**A**), respiratory rate (RR) (**B**), skin temperature (ST) (**C**), and rectal temperature (RT) (**D**) among each group at 15:00 on Day 4. Bars with different lowercase letters above their whiskers differ significantly (*p* < 0.05), while those with the same or no lowercase letters means no significant difference. C, MHS, SHS, and RHS represent the control group, the moderate heat stress group, the sever heat stress group, and the relief group under serve heat stress.

In the MHS group, the RR increased significantly on Day 4, compared to Day 3 and 5 (*p* < 0.05). While in the SHS group, the HR and ST on the 4th day were also highest, compared with Day 3 and Day 5 of the experiment (*p* < 0.05). Moreover, both in the MHS and SHS groups, the HR, RR, ST, and RT increased initially and then decreased as time went on (Table S3). This suggested that the HS mainly played a critical role in the physiological indices of lambs on Day 4. Results showed that on Day 4 of the experiment, the RR, ST, and RT in the MHS, SHS and RHS groups were highest at 15:00, compared with the other three time points (Figure S2), which indicated that the target temperature in each treatment group indeed had an important effect on the lambs. Based on this evidence, the difference of HR, RR, ST and RT were compared among each group at 15:00 on Day 4. The RR and ST in the SHS groups were markedly higher than in the MHS and RHS groups, which were both dramatically higher when compared with the C group (Figure 1). These results verified that the adverse effects can be exacerbated with an increased HS level, and that the supplement of folic acid and taurine can significantly relieve the detrimental effect induced by HS.

### 3.3. The Folic Acid and Taurine Alleviate the Deleterious Effects of HS on Oxidative Stability and Immune Status in Dorper–Hu Crossbred Lambs

The antioxidative parameters were affected significantly by the day or treatment or day and treatment. The concentration of T-AOC and GPx were lowest on the 4th day, compared with the 1st day and 7th day; concomitantly, the MDA increased notably on the 4th day (*p* < 0.05) (Table 1). All of which can illustrate that a deleterious effect on the antioxidant capacity of HS was triggered on the 4th day. In addition, the SOD and GPx decreased significantly in the SHS group compared with the C group, while the content of MDA was highest in the SHS group, compared to other groups (*p* < 0.05). The content of SOD and GPx in the RHS group was much higher than in the MHS and SHS groups (*p* < 0.05). These results manifested that folic acid and taurine may ameliorate the oxidative damage under HS.

Similar to the anti-oxidative indices, the day or treatment had a significant effect on inflammatory cytokines, immunoglobins, and the heat shock protein (*p* < 0.05) (Table 2). The TNF-α, IL-13, IgA, IgG, and IgM in serum were increased signally on Day 4 and Day 7, compared to the first day (*p* < 0.05), while these immune factors were increased slightly on the 4th day, compared to the 7th day. The content of IL-1βand HSP70 in serum were highest on the 4th day, compared to Day 1 and Day 7 (*p* < 0.05), despite there being a higher concentration on the 7th day, compared to the first day of the experiment (*p* < 0.05). These evidenced that the HS mainly exerted an unfavorable influence on the immunity on the 4th day. Moreover, lambs may exhibit a good adaptation to HS under the conditions of this test. As shown in Table 2, compared with the C group, the level of TNF-α and HSP70 was significantly increased in the SHS group, as well as IL-13, IgA, IgG, and IgM being notably decreased (*p* < 0.05). Concomitantly, the content of IL-10, IL-13, IgA, IgG, and IgM in the RHS group was significantly higher than in the SHS group, while there was an opposite trend for TNF-α and HSP70 between these two groups (*p* < 0.05). Compared with the C group, the TNF-α and HSP70 also increased dramatically in the MHS group, while the IgA and IgG showed a marked decrease (*p* < 0.05). As for the TNF-α, it increased significantly in the SHS group, compared to the MHS group, while there was an inverse tendency for the IgA and IgG (*p* < 0.05). Also, the day and treatment simultaneously had an important impact on the content of IgG (*p* < 0.05) (Table 2). These results indicated that HS, and especially severe HS, can inhibit the immunity of lambs via upregulating the proinflammatory factor or downregulating the anti-inflammatory cytokine and the immunoglobin; furthermore, the supplement of folic acid and taurine can relieve the adverse effect on immunity of HS.

### 3.4. Effects of Folic Acid and Taurine on Rumen Fermentation of Dorper-Hu Crossed Sheep under HS

As depicted above, it was mainly on the 4th day that the HS had a deleterious effect on physiological indices, immunity and antioxidant capacity. Hereby, we focused on exploring the difference of rumen ferment parameters among each group on Day 4. As shown in Table 3, there were no significant difference of pH and the total volatile fatty acid (TVFA). Compared with the C group, the content of acetate increased significantly in the SHS and RHS groups (*p* < 0.05), while there was no significant difference between the two groups. The percent of propionate was higher in the SHS and RHS groups than in the MHS group (*p* < 0.05), and there was no significant difference compared to the C group. Compared with the C group, the ratio of Acetate/Propionate (A/P) increased markedly in the SHS group and decreased significantly in the RHS group (*p* < 0.05). The proportion of isobutyrate and valerate in the SHS and RHS groups was higher than in the C and MHS groups (*p* < 0.05). In addition, the concentration of NH_3_-N was lowest in the MHS group, compared with the C, SHS, and RHS groups.

### 3.5. Comparison of Bacterial Community Composition in Rumen among Each Group

**Figure 2:**
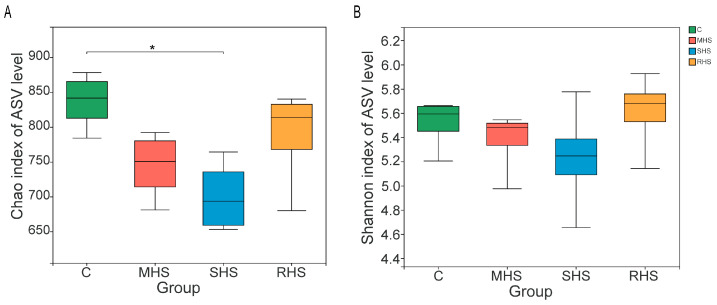
Alpha diversity of the bacterial community within each group on the 4th day. The richness and diversity were calculated via Chao (**A**) and Shannon (**B**) indexes, respectively. Bars with a star symbol above their whiskers are significantly different among each group. “*” means “0.01 < *p* < 0.05”.

A total of 987,738 valid sequences were obtained from twenty-four samples of ruminal digesta collected from four groups on the 4th day of the experiment. After denoised, there were 697,223 sequences with an average of (29,050.96 ± 3872.99) sequences per sample (Table S4). The rarefaction curves approached a plateau and the average Good’s coverage of all samples was one, indicating the sequencing depth was sufficient to precisely describe the microbial composition of each sample (Figure S3; Table S5). Based on taxonomic assignment of ASVs, 5861 ASVs, or 276 genera belonging to 21 phyla, were identified in all samples. The Chao value of bacterial community in the SHS group decreased significantly, compared with the C group (*p* < 0.05). The Chao and Shannon value of bacterial community decreased with the aggravation of HS, and then increased somewhat after folic acid and taurine were added (Figure 2). The analysis of α diversity revealed that the addition of folic acid and taurine to the dietcan prevented the reduction of bacterial richness and diversity in rumen induced by HS. The analysis of PCoA and ANOSIM showed that there was no evident distinction of samples among each group (Figure S4), implying the HS had no significant influence on the composition of bacterial community.

**Figure 3:**
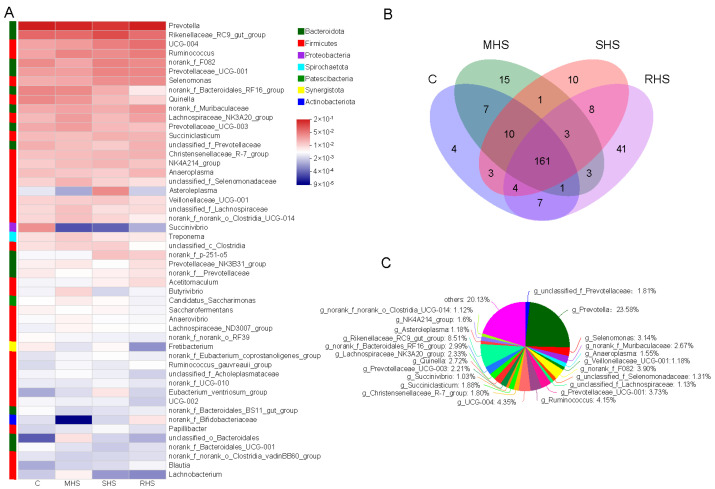
The composition of bacterial communbity (at the genus level) in runimal digesta. (**A**) Bacterial taxa composition in each group (the top 50 genera). (**B**) Venn diagram of bacterial genera shared among each group. (**C**) The proportion of the top 23 shared bacterial genera in each group (the average relative abundance ≥1%).

**Figure 4:**
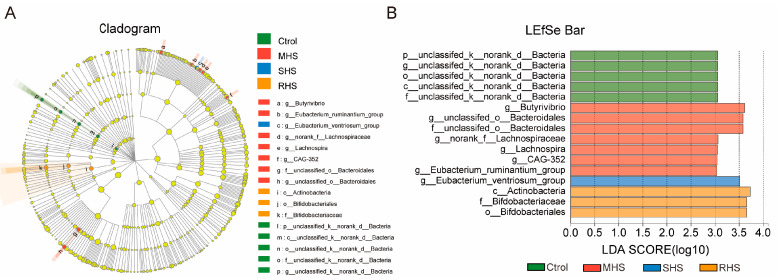
The linear discriminant analysis effect size (LEfSe) analysis (**A**) and the LDA score (**B**). The color represents the group, different color dots represent the significant difference of the corresponding group of species (important groups), yellow dots represent no significant difference groups, the circles represent the phylum, order, family and genus in order, from inside to outside. The length of the bar graph represents the LDA value (magnitude of variability), mainly showing the colonies with statistically significant differences (LDA > 3).

The majority of the sequences belonged to the phylum Bacteroidota (53.37%), Firmicutes (41.37%), Proteobacteria (1.30%), and Spirochaetota (1.27%) (Table S6). As for these predominant bacterial phyla, there was no significant difference among each group. Compared with the C group, the proportion of the Bacteroidota decreased 7.99% and 8.82% in the MHS and SHS groups, respectively; while the abundance of the Firmicutes in these two groups increased 24.96% and 26.83%, respectively. There was a little increase of the Bacteroidota or a decrease of Firmicutes after adding the folic acid and taurine. At the genus level, 281 genera were found and the top 50 bacterial taxa were identified, as shown in Figure 3A. According to the Venn diagram analysis, 161 genera were found to be shared among the different groups (Figure 3B). In these common genera, 23 core genera were selected to elucidate the effect of HS on bacterial community, based on a criterion of those with the average proportion ≥1% that was based on all common genera (Figure 3C). As the first predominant bacterial genus, the genus *Prevotella* was lowest in the SHS group, while the genera *Rikenellaceae_RC9_gut_group* and *Asteroleplasma* in the SHS group were notably higher than in the other three groups (*p* < 0.05). Compared with Group C and the RHS group, the genus *Quinella* and *Succinivibrio* decreased dramatically in the MHS and SHS group (*p* < 0.05). The proportion of *norank_f_Bacteroidales_RF16_group* were higher in the C and MHS groups than in the SHS and RHS groups (*p* < 0.05). These results showed that the HS had significant influence on ruminal core bacterial genera, and that several decreased bacterial taxa triggered by HS can recover somewhat after the folic acid and taurine is added (Table 4). Based on the LEfSe analysis, compared to the C group, the mainly marked taxa in MHS group included the genus *Butyrivibrio*, *norank_f_Lachnospiraceae*, *Lachnospira*, and *CAG-352*; in the SHS group, the marked taxa consisted of *Eubacterium_ventriosum_group*, while the c_Actinobacteria and f_Bifidobacteriaceae were mainly enriched in the RHS group (Figure 4).

### 3.6. Correlation Analysis between the Predominant Genus and the Ruminal Ferment Parameters, the Immune Cytokines or Antioxidant Indices

**Figure 5:**
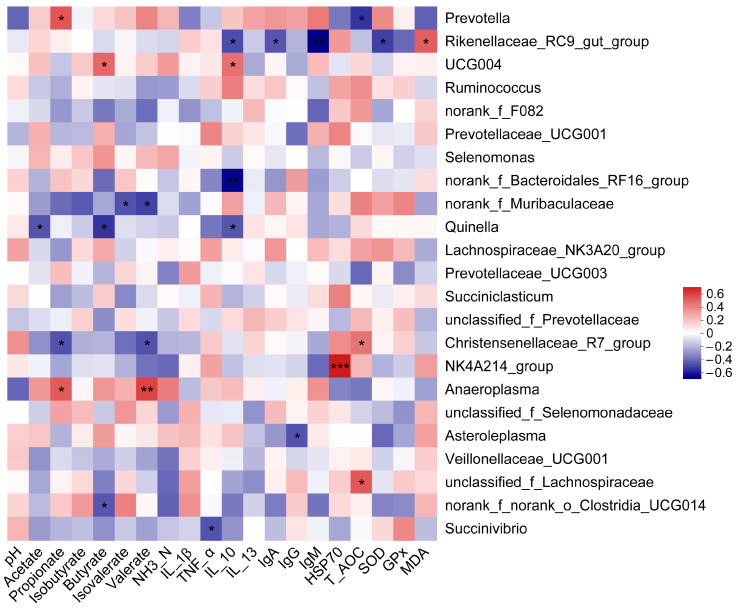
Correlation heatmap (R > 0.4) between the predominant common bacterial genera and the pH, VFA, inflammatory cytokine, immunoglobin, HSP70, T-AOC, SOD, GPx, and MDA. The blue and red squares represent the positive and negative correlation, respectively. The shade of color indicates the level of correlation. * *p* < 0.05, ** 0.001 < *p* < 0.01, *** *p* < 0.001.

The environmental factors, i.e., the pH, VFA, inflammatory cytokine, immunoglobin, HSP70, T-AOC, SOD, GPx, and MDA, were selected to perform a correlation analysis with 23 dominant bacterial genera by calculating the Sperman’s correlation coefficient (R > 0.4, *p* < 0.001). The correlation heatmap results showed that the genus *Prevotella* was positively correlated with propionate but exhibited a negative correlation with T-AOC. The genus *Rikenellaceae_RC9_gut_group* was positively associated with MDA, while it was negatively involved in IL-10, IgA, IgM, and SOD. The genus *UCG004* was positively related to butyrate and IL-10, while *norank_f_Bacteroidales_RF16_group* negatively participated in IL-10. The genus *norank_f_Muribaculaceae* was negatively correlated with isovalerate and valerate, and there was also a negative relationship between the *Quinella* and the acetate, butyrate and IL-10. The genus *Christensenellaceae_R7_group* was positively correlated with T-AOC; nevertheless, it was negatively involved in propionate and valerate. The genus *NK4A214_group* displayed an extremely positive relation to HSP70, while the genus *Asteroleplasma* was negatively related to the IgG. The genus *Anaeroplasma* was positively associated with the propionate and valerate, while the genus *Succinivibrio* was negatively involved in TNF-α. The genus *unclassified_f_Lachnospiraceae* was positively correlated with the T-AOC (Figure 5).

## 4. Discussion

The object of this study was to explore the detrimental effects of different HS levels on growth performance, physiological indices, immune status, antioxidative capacity, rumen microbial composition and fermentation, and to investigate if the supplementation of folic acid and taurine can mitigate the adverse influence induced by HS. In this study, the folic acid and taurine played a protective role in alleviating HS-induced decreased ADG. To some extent, they can also relieve the decrease of ADFI caused by HS. Likewise, two studies have reported that folic acid or taurine can improve poultry performance in terms of live BW, ADG, and feed efficiency under HS conditions [14,19]. It is well known that the adverse effect of HS on animal growth performance is partly due to the decrease of feed intake [3,11,32]. The reduction of feed intake was a natural, protective, and adaptive mechanism of ruminants under high ambient temperature to counteract the rise in metabolic heat production, which is mainly attributed to feed fermentation in rumen and may compromise their thermoregulation [2]. It is accepted that mild to severe HS can increase the metabolic maintenance requirement by 7 to 25% [5]; however, the remaining energy for production will decrease when the energy is consumed to maintain the body temperature increases. Along with the decrease of energy obtained due to reduced feed intake, an energy deficiency may occur in cows under HS [10,33]. Physiological indices, such as RR, HR, ST, and RT, are the primary coping responses to both the level and duration of HS. Previous studies demonstrated the susceptibility of sheep to HS can be verified by higher RR, RT, or ST, when exposed to HS [3,34]. These findings are in line with the results obtained in our study, which indicated that HS increased RR and ST, compared to the C group. It was suggested that the physiological disorders caused by HS may be the inducing factor of the rise of RR and ST, which may provoke inflammation [4]. Intriguingly, the supplementation of folic acid and taurine evidently inhibited the increase of RR and ST induced by HS, which implied that they may play an important role in preventing the occurrence of inflammation.

Generally, the antioxidant enzyme system is considered to be the first barrier of antioxidative defense in animals, which is mainly composed of SOD, GPx, and catalase [9]. HS can repress the activity of mitochondria to increase the production of reactive oxygen species (ROS)/free radicals, which disrupts the dynamic balance of redox and induces oxidative stress [12,35]. And the excess ROS induced the oxidative damage to DNA, lipid, and protein, and can ultimately result in the death of cell and tissue. In addition, there is evidence that animals exhibit a poor antioxidant capacity when exposed to chronic HS [36,37]. In this experiment, HS significantly reduced the activity of SOD and GPx in serum but significantly increased MDA concentration, which was consistent with the above-mentioned findings. Noteworthily, the addition of folic acid and taurine signally elevated the oxidative capacity of lambs under HS. Studies of the effects of folic acid or taurine on broilers when exposed to HS confirmed our results, which may be ascribed to the activity of eliminating free radicals belonging to folic acid and taurine [14,22,23]. In addition, compared with the first day and 7th day, the T-AOC, SOD, and GPx were significantly decreased, whereas the content of MDA was highest on the 4th day, which suggested that HS may have a deleterious effect on antioxidative capacity on the 4th day.

It is well known that the immune response is one of the most important mechanisms evolved to defend against environmental stressors. HS can have a negative influence on immune system via altering the balance of T-helper 1 (Th1) and T-helper 2 (Th2), which may contribute to animals becoming more susceptible to diseases. To the best of our knowledge, the Th1 and Th2 are responsible for producing proinflammatory and anti-inflammatory response, respectively [38,39]. In this experiment, compared with the C group, HS significantly elevated the concentration of proinflammatory cytokines (TNF-α) and induced the evident decrease of anti-inflammatory cytokines (IL-10 and IL-13), which may be attributed to the upregulation of Th1:Th2. Moreover, the content of HSP70 in serum increased significantly in the MHS and SHS groups. These results were in line with a report conducted by Ashgan M. et al., who considered that HS enhanced the release of serum proinflammatory cytokines and HSP 70 [40]. It has been established that extracellular HSPs released by injured or dying cells, including HSP 70, can act as damage-associated molecules to activate the surfaceTLR4 of dendritic cells and further generate a proinflammatory reaction [41]. As can be seen above, the oxidative stress induced by HS can lead to cell damage and death. Dietary supplementation of folic acid and taurine decreased the proinflammatory cytokines and HSP 70, and increased the anti-inflammatory cytokines; this suggested that the folic acid and taurine exhibited an anti-inflammatory effect. The measurement of serum immunoglobulin is a method to evaluate humoral immunity. Poultry exposed to HS had a reduction of IgA, IgG and IgM [42]. Another study of calves also showed that there was a reduced serum IgG level under HS, compared to calves in a cool condition [43]. These results are consistent with our findings, which implied that HS play an adverse role in humoral immune function of lambs. Folic acid and taurine significantly increased the IgA, IgG and IgM contents in serum. These findings suggested that the immunoregulatory effect of folic acid and taurine in the current study was mainly ascribed to their antioxidant and anti-inflammatory properties. In addition, as the experiment went on, the indices of MDA, IL-1β, TNF-α, and HSP 70 were highest on the 4th day. The contents of IL-10, IL-13, IgA, IgG, and IgM significantly increased at first and then decreased, whereas the T-AOC and GPx showed an opposite trend, which all revealed that HS occurred mainly on the 4th day and there may be an adaptation to HS for lambs, with time.

Actually, there is evidence that HS can induce the disruption of the integrity of the gastrointestinal barrier, which allows the penetration of LPS into the blood and recruits distinct immune cell populations into the bovine intestine, resulting in the occurrence of inflammation [6,44]. For ruminants, ruminal microbial composition and fermentation played vital roles in maintaining the structural and functional integrity of the gastrointestinal barrier. Generally, the microbial fermentative capacity in rumen can be evaluated via monitoring the variation of pH, VFA, and NH_3_-N. To the best of our knowledge, there was an increase of TVFA with the exacerbation of HS, along with a decrease of pH, which was consistent with a previous study [45]. The increase of TVFA was one of the most crucial factors contributing to the decrease of pH. As we all know, VFA, as the main energy source for sheep, is extremely vital to the growth and development of sheep. Moreover, compared with the C group, there was a significant increase of acetate and decrease of propionate in the SHS group. And the ratio of acetate/propionate increased significantly in SHS group, compared to other groups. The increase of acetate/propionate ratio usually means a decrease of fermentation efficiency, which may have an unfavorable effect on the development of muscle and adipose deposition for mutton sheep. A study of goats perceived that HS reduced the concentration of NH_3_-N [46]. Similar to this finding, the content of NH_3_-N was decreased markedly by HS, which may be due to the imbalance of the proteolytic bacteria and NPN-utilizing bacteria caused by the high ambient temperature. The decrease of pH can also confirm this, because a variety of bacteria that were susceptible to the low pH can be repressed [47,48]. However, the supplementation of folic acid and taurine significantly decreased the ratio of acetate/propionate and elevated the concentration of NH_3_-N, compared with the MHS and SHS groups, which mirrored the observation that the folic acid and taurine can effectively protect the microbial fermentation in rumen from the deleterious influence of HS.

To some extent, rumen fermentation was a response to variations of microbiota in rumen [45]. It has been established that HS can induce an imbalance between beneficial and pathogenic bacteria in the intestinal tract of poultry, which may cause metabolic disturbance of microbial community [12]. In our study, HS significantly reduced the Chao and Shannon index, which reflects the richness and diversity of microbiota, respectively. A study of the effect of HS on the cecal microbiota in broilers obtained a similar finding [49]. But several studies reported that there was no significant effect of HS on the α-diversity of microbiota in the rumen or gut [45,50]. The difference of results may be ascribed to the ambient temperature, duration, species, ages, or segments of gastrointestinal tract. Interestingly, the supplementation of folic acid and taurine restored the richness and diversity of bacterial community under HS. Contrary to the results of α diversity, β-diversity based on PCoA showed no significant changes in the bacterial community composition and structure. This suggested that HS may not thoroughly disrupt the ruminal microbial structure under this experimental condition. Results showed that the phyla Bacteroidota and Firmicutes were the two most predominant bacteria taxa, and to some extent HS increased the proportion of Firmicutes and decreased the abundance of Bacteroidota. The increase of Firmicutes to Bacteroidetes ratio can exacerbate negative energy balance, despite it possibly promoting the extraction of energy from feed and facilitating adipogenesis [51]. In this study, as the first dominant bacterial genus, *Prevotella*, which can perform variable physiological functions in rumen such as digest carbohydrates and proteins [52], was lowest in the SHS group. This may explain the significant decrease of NH_3_-N under HS. As an acetate- and propionate- producing bacteria [53], *Rikenellaceae_RC9_gut_group* was highest in the SHS group, and it showed a positive correlation with MDA and a negative relation with IL-10, IgA, IgM, and SOD under this experimental condition. We assumed that the genus *Rikenellaceae_RC9_gut_group* may be conducive to HS responses in sheep by elevating ruminal heat production. As reported earlier, *Rikenellaceae_RC9_gut_group* can degrade the carbohydrates to VFAs for energy supply, so as to protect the body protein decomposing to amino acid to gluconeogenesis, due to the reduced feed intake induced by HS [54]. Previous studies have reported that the genus *Asteroleplasma*, as a potential pathogenic bacteria, was associated with intestinal inflammation, and that chronic HS can induce the disorder of gut immune function in growing pigs via increasing its abundance [55,56]. Similar to these findings, HS led to the increase of Asteroleplasma and i was negatively associated with IgG in this study, which implied that HS may inhibit the immune function of lambs by altering the composition of the bacterial community. In addition, HS reduced significantly the proportion of *Quinella* and *Succinivibrio* when compared with Group C. It is well known that the genus *Quinella* is an iconic uncultured bacteria in rumen, which mainly participates in lactate and propionate formation and is often found in rumen of sheep that emit low CH_4_ production [57]. And, at present, it was negatively involved in the formation of acetate and butyrate. Consistent with our results, *Succinivibrio* reduced in HS-challenged pigs [58]. As a major propionate producing genera, *Succinivibrio* is not only positively correlated with feed efficiency in sheep [59], but also plays a protective role in maintaining the integrity of gut mucosal barrier in pigs via promoting the expression of occluding and claudin-3 [58]. In this study, *Succinivibrio* was also negatively associated with TNF-α, which indicated that it may inhibit the inflammation of lambs. Moreover, several marked bacterial taxa, such as *Butyrivibrio* and *Eubacterium_ventriosum_group*, were enriched in the MHS and SHS group, respectively. *Butyrivibrio* can utilize the cellulose, hemicelluloses, and pectin to generate butyrate, which is responsible for the proliferation and differentiation of the epithelial cell in the intestine and can modulate the inflammatory response. Hence, we assumed that *Butyrivibrio* may aid in restoring the rumen homeostasis of heat-stressed lambs under the mild/middle level of HS. A previous study of the effect of HS on the intestinal barrier, inflammatory signals, and microbiome communities in dairy calves obtained similar results [60]. As for *Eubacterium_ventriosum_group*, it was associated with a risk of IBD [61], which indicated that harmful bacteria increased under severe HS. *Bifidobacterium*, as a representative of the well-known probiotic family of f_Bifidobacteriaceae, play various beneficial roles in the preserving host’s health, including carbohydrate degradation, immunomodulation, and protection from pathogens [62]. The marked bacteria in the RHS group mainly consisted of f_Bifidobacteriaceae, which suggested that the supplementation of folic acid and taurine under severe HS may provide a protective effect for lambs via increasing the proportion of beneficial bacteria in rumen.

## 5. Conclusions

Taken together, HS affects the growth performance of Dorper-Hu crossbred lambs regarding a decrease in ADG and ADFI. Additionally, HS exerts an adverse influence on the physiological indices, oxidation resistance, immunomodulation ability, and rumen fermentation of Dorper-Hu crossbred lambs on the 4th day by increasing RR and ST, increasing the concentration of MDA, reducing the T-AOC, SOD, and GPx in serum, mounting the contents of TNF-α and IL-1β, decreasing IL-10, IL-13, IgA, IgG, and IgM, and boosting the A/P ratio. Additionally, an adaptation to HS may be possessed by lambs. More importantly, the supplementation of folic acid and taurine can effectively ameliorate HS-induced pathology on mentioned body characteristics via enhancing/restoring the abundance of beneficial bacteria, such as *Prevotella*, *Quinella*, *Succinivibrio*, and f_Bifidobacteriaceae, and simultaneously decreasing the proportion of pathogen bacteria (*Asteroleplasma* and *Eubacterium_ventriosum_group*). Therefore, the addition of folic acid and taurine can be an effective measure to mitigate the unfavorable effects of HS on sheep.

## Figures and Tables

**Table 1:** Comparison of antioxidant parameters among each group on the 1st, 4th and 7th day.

Items	Day	Treatment (T)					*p*		
		C	MHS	SHS	RHS	D	T	D	T × D
T-AOC/(mM/mL)	1 d	0.40 ± 0.02	0.41 ± 0.02	0.38 ± 0.01	0.46 ± 0.05	0.42 ± 0.02 ^AB^	0.193	0.001	0.113
	4 d	0.35 ± 0.05	0.34 ± 0.10	0.27 ± 0.05	0.44 ± 0.13	0.38 ± 0.03 ^A^			
	7 d	0.52 ± 0.08	0.48 ± 0.06	0.39 ± 0.08	0.47 ± 0.09	0.46 ± 0.02 ^B^			
	T	0.42 ± 0.04	0.41 ± 0.04	0.33 ± 0.04	0.46 ± 0.04				
SOD/ (U/mL)	1 d	31.55 ± 2.09	30.48 ± 2.21	29.00 ± 1.07	43.13 ± 0.95	33.65 ± 1.04	0.025	0.199	0.090
	4 d	37.49 ± 2.55	35.07 ± 3.61	22.90 ± 1.63	42.04 ± 2.33	32.76 ± 2.68			
	7 d	38.32 ± 1.79	37.09 ± 1.21	31.59 ± 1.15	37.38 ± 3.53	36.89 ± 1.38			
	T	35.64 ± 2.29 ^b^	33.91 ± 2.96 ^ab^	27.83 ± 2.09 ^a^	40.35 ± 2.56 ^b^				
GPx/ (U/mL)	1 d	68.61 ± 2.49	62.22 ± 4.86	52.59 ± 3.41	73.17 ± 2.76	64.10 ± 2.03 ^BC^	<0.001	<0.001	0.767
	4 d	58.89 ± 4.73	44.17 ± 2.23	37.33 ± 1.72	67.28 ± 8.01	52.44 ± 2.94 ^A^			
	7 d	71.14 ± 4.27	68.86 ± 5.18	60.86 ± 7.15	83.54 ± 8.12	72.22 ± 3.34 ^C^			
	T	65.92 ± 3.18 ^bc^	57.80 ± 3.18 ^ab^	50.40 ± 3.56 ^a^	74.67 ± 2.91 ^c^				
MDA/ (nmol/mL)	1 d	1.27 ± 0.07	1.77 ± 0.33	4.78 ± 0.85	2.01 ± 0.18	2.53 ± 0.26 ^A^	<0.001	0.016	0.205
	4 d	2.41 ± 0.34	3.81 ± 0.51	6.23 ± 0.68	2.08 ± 0.30	2.79 ± 0.26 ^B^			
	7 d	2.38 ± 0.11	2.42 ± 0.0.12	2.90 ± 0.74	2.31 ± 0.15	2.61 ± 0.29 ^A^			
	T	2.15 ± 0.17 ^a^	2.65 ± 0.15 ^a^	4.42 ± 0.17 ^b^	2.14 ± 0.12 ^a^				

D and T represents the day of the experiment and treatment, respectively; Mean values with different superscripted lowercase letters within the same row or capital letters within the same column differ significantly (*p* < 0.05), while the mean values with no letters or with the same letters means no significant difference.

**Table 2:** Comparison of inflammatory cytokine, immunoglobin, and the heat shock protein among each group on the 1st, 4th and 7th day.

Items	Day	Treatment (T)				D	*p*		
		C	MHS	SHS	RHS		T	D	T × D
IL-1β /(pg/mL)	1 d	55.26 ± 8.46	53.27 ± 5.36	63.79 ± 5.09	60.12 ± 5.93	58.11 ± 1.63 ^A^	0.226	<0.001	0.167
	4 d	79.66 ± 5.32	88.78 ± 3.90	91.03 ± 4.01	74.00 ± 7.50	83.34 ± 1.98 ^C^			
	7 d	67.63 ± 7.25	72.64 ± 4.48	67.53 ± 4.92	68.60 ± 9.00	69.10 ± 2.60 ^B^			
	T	67.52 ± 2.18	71.56 ± 2.18	74.12 ± 2.81	67.57 ± 2.43				
TNF-α/ (pg/mL)	1 d	69.81 ± 3.14	79.65 ± 2.59	104.51 ± 2.85	79.20 ± 3.16	83.29 ± 0.91 ^A^	<0.001	<0.001	0.250
	4 d	77.13 ± 8.10	127.63 ± 8.26	135.17 ± 5.64	125.79 ± 8.92	116.43 ± 5.56 ^B^			
	7 d	97.46 ± 7.40	115.86 ± 6.89	137.13 ± 13.34	104.18 ± 8.38	113.66 ± 4.73 ^B^			
	T	81.47 ± 3.37 ^a^	107.71 ± 3.37 ^b^	125.60 ± 4.13 ^c^	103.06 ± 3.37 ^b^				
IL-10/ (pg/mL)	1 d	84.81 ± 6.57	91.52 ± 4.89	75.56 ± 4.84	124.16 ± 6.74	94.01 ± 2.60 ^A^	<0.001	<0.001	0.120
	4 d	89.67 ± 7.77	96.72 ± 7.56	100.80 ± 9.23	135.20 ± 5.86	105.60 ± 3.50 ^B^			
	7 d	108.20 ± 8.79	123.84 ± 4.63	121.12 ± 7.45	129.33 ± 6.12	120.62 ± 3.74 ^C^			
	T	94.23 ± 2.89 ^a^	104.03 ± 3.16 ^b^	99.16 ± 2.89 ^ab^	129.56 ± 3.16 ^c^				
IL-13/ (pg/mL)	1 d	361.24 ± 21.23	340.48 ± 38.38	304.73 ± 40.98	406.31 ± 54.91	353.19 ± 24.34 ^A^	0.042	<0.001	0.785
	4 d	602.60 ± 44.28	542.76 ± 32.42	521.95 ± 17.11	617.91 ± 44.75	571.30 ± 20.80 ^B^			
	7 d	607.28 ± 14.56	540.80 ± 42.85	453.61 ± 43.03	513.39 ± 45.26	528.77 ± 21.74 ^B^			
	T	523.71 ± 26.03 ^b^	474.68 ± 23.28 ^ab^	426.77 ± 23.28 ^a^	512.53 ± 21.25 ^b^				
IgA/(μg/mL)	1 d	207.61 ± 12.08	154.42 ± 14.61	130.79 ± 11.82	223.94 ± 14.78	179.19 ± 4.98 ^A^	<0.001	<0.001	0.220
	4 d	266.54 ± 19.24	270.91 ± 14.64	213.09 ± 7.25	308.69 ± 11.81	264.81 ± 7.21 ^B^			
	7 d	248.40 ± 14.94	241.45 ± 13.97	218.31 ± 16.63	254.13 ± 16.61	240.57 ± 8.40 ^B^			
	T	240.85 ± 5.80 ^c^	222.26 ± 5.29 ^b^	187.40 ± 6.48 ^a^	262.25 ± 5.29 ^d^				
IgG/(mg/mL)	1 d	61.30 ± 2.72 ^de^	47.19 ± 4.07 ^ab^	42.40 ± 0.89 ^a^	51.51 ± 2.69 ^bc^	50.60 ± 1.38 ^A^	<0.001	<0.001	0.017
	4d	70.93 ± 3.40 ^f^	68.86 ± 2.89 ^ef^	49.55 ± 2.69 ^bc^	67.32 ± 1.95 ^ef^	64.16 ± 1.41 ^B^			
	7 d	66.51 ± 3.19 ^ef^	57.01 ± 2.03 ^cd^	59.40 ± 3.46 ^cd^	62.40 ± 2.35 ^def^	61.33 ± 1.39 ^B^			
	T	66.24 ± 1.54 ^c^	57.68 ± 1.54 ^b^	50.45 ± 1.69 ^a^	60.41 ± 1.54 ^b^				
IgM/(μg/mL)	1 d	1325.72 ± 85.89	1013.81 ± 94.65	1040.96 ± 77.88	1469.13 ± 40.96	1212.41 ± 40.35 ^A^	<0.001	<0.001	0.141
	4 d	1722.56 ± 31.73	1871.63 ± 79.66	1395.73 ± 123.40	2202.52 ± 150.60	1798.11 ± 54.57 ^B^			
	7 d	1752.58 ± 123.82	1732.44 ± 135.26	1671.87 ± 113.25	2009.63 ± 102.85	1791.63 ± 66.88 ^B^			
	T	1600.29 ± 70.54 ^b^	1539.29 ± 63.09 ^ab^	1369.52 ± 63.09 ^a^	1893.76 ± 63.09 ^c^				
HSP70/(pg/mL)	1 d	419.53 ± 17.03	388.31 ± 27.83	453.52 ± 23.29	436.67 ± 13.07	424.51 ± 12.10 ^A^	0.021	0.001	0.170
	4 d	477.27 ± 23.62	695.14 ± 25.84	667.88 ± 35.55	594.80 ± 25.38	608.77 ± 19.09 ^C^			
	7 d	480.89 ± 36.95	532.87 ± 16.72	571.91 ± 22.04	524.59 ± 30.75	527.57 ± 15.41 ^B^			
	T	459.23 ± 27.37 ^a^	538.77 ± 19.35 ^b^	564.44 ± 26.37 ^b^	518.67 ± 20.54 ^b^				

D and T represent the day of the experiment and treatment, respectively; Mean values with different superscripted lowercase letters within the same row or capital letters within the same column differ significantly (*p* < 0.05), while the mean values with no letters or with the same letters mean no significant difference.

**Table 3:** Comparison of pH, VFA and NH3-N in rumen liquid of sheep in each group.

Items	C	MHS	SHS	RHS	*p*
pH	6.67 ± 0.05	6.61 ± 0.06	6.44 ± 0.09	6.38 ± 0.15	0.140
TVFA/(mmol/L)	78.93 ± 3.38	91.98 ± 2.60	105.29 ± 7.53	99.05 ± 11.26	0.085
Acetate/(%)	65.68 ± 1.05 ^ab^	63.65 ± 0.50 ^a^	67.71 ± 0.71 ^b^	66.72 ± 1.15 ^b^	0.026
Propionate/(%)	15.36 ± 0.75 ^ab^	17.04 ± 0.81 ^b^	13.07 ± 0.86 ^a^	12.88 ± 0.84 ^a^	0.005
Isobutyrate/(%)	0.82 ± 0.04 ^b^	0.81 ± 0.04 ^b^	0.66 ± 0.03 ^a^	0.65 ± 0.05 ^a^	0.012
Butyrate/(%)	15.60 ± 1.69	15.84 ± 0.95	16.48 ± 0.93	17.75 ± 0.69	0.548
Isovalerate/(%)	1.23 ± 0.10 ^bc^	1.39 ± 0.09 ^c^	1.05 ± 0.07 ^ab^	0.96 ± 0.05 ^a^	0.006
Valerate/(%)	1.32 ± 0.07 ^b^	1.26 ± 0.08 ^b^	1.04 ± 0.05 ^a^	1.04 ± 0.08 ^a^	0.018
Acetate/Propionate	4.32 ± 0.19 ^b^	3.78 ± 0.20 ^b^	5.30 ± 0.36 ^c^	2.47 ± 0.19 ^a^	<0.001
NH_3_-N/(mmol/L)	16.71 ± 0.95 ^bc^	12.64 ± 1.05 ^a^	15.63 ± 1.20 ^b^	19.11 ± 1.08 ^c^	0.004

Mean values with different superscripted lowercase letters within the same row differ significantly (*p* < 0.05), while the mean values with no letters or with the same superscripted lowercase letters means no significant difference.

**Table 4:** The comparison of predominant bacterial genus (the average relative abundance ≥1%) among each group (%).

Species Name	Group				SEM	*p*
	Control	MHS	SHS	RHS		
*Prevotella*	26.689	23.358	17.833	25.994	1.817	0.312
*Rikenellaceae_RC9_gut_group*	7.384 ^a^	7.154 ^a^	12.290 ^b^	6.729 ^a^	0.794	0.006
*UCG004*	2.784	3.457	5.139	6.530	0.703	0.593
*Ruminococcus*	2.503	4.900	3.998	5.195	0.623	0.442
*norank_f_F082*	4.011	2.477	4.854	4.107	0.377	0.150
*Prevotellaceae_UCG001*	2.899	2.980	4.739	4.518	0.429	0.281
*Selenomonas*	2.257	2.517	3.797	4.036	0.638	0.715
*norank_f_Bacteroidales_RF16_group*	4.267 ^b^	3.945 ^b^	2.184 ^a^	0.737 ^a^	0.732	0.044
*norank_f_Muribaculaceae*	2.553	2.720	2.263	3.227	0.351	0.516
*Quinella*	4.351 ^c^	1.781 ^ab^	1.122 ^a^	3.139 ^b^	0.515	0.039
*Lachnospiraceae_NK3A20_group*	1.826	3.209	1.778	2.790	0.244	0.439
*Prevotellaceae_UCG003*	2.597	3.032	1.840	1.580	0.177	0.584
*Succiniclasticum*	1.701	1.595	2.228	1.961	0.243	0.192
*unclassified_f_Prevotellaceae*	2.434	1.597	1.284	2.076	0.223	0.599
*Christensenellaceae_R7_group*	1.474	1.632	1.893	2.095	0.176	0.634
*NK4A214_group*	1.253	1.690	1.968	1.474	0.124	0.211
*Anaeroplasma*	1.630	1.347	1.273	1.949	0.169	0.736
*unclassified_f_Selenomonadaceae*	1.153	1.997	1.128	0.888	0.220	0.294
*Asteroleplasma*	0.362 ^a^	0.138 ^a^	4.173 ^b^	0.220 ^a^	0.071	0.027
*Veillonellaceae_UCG001*	1.037	1.266	1.395	0.925	0.145	0.424
*unclassified_f_Lachnospiraceae*	1.025	1.553	0.903	0.972	0.130	0.283
*norank_f_norank_o_Clostridia_UCG014*	1.081	1.499	1.091	0.684	0.137	0.220
*Succinivibrio*	3.837 ^b^	0.030 ^a^	0.042 ^a^	2.138 ^b^	0.809	0.006

Mean values with different superscripted lowercase letters within the same row differ significantly (*p* < 0.05), while the mean values with no letters or with the same superscripted lowercase letters mean no significant difference.

## Data Availability

The 16S rRNA data of digesta samples in rumen are available from the National Center for Biotechnology Information (NCBI) under accession PRJNA1072299.

## Supplementary Materials

The following supporting information can be downloaded at: https://www.mdpi.com/article/10.3390/ani14070998/s1, Figure S1: Average daily temperature humidity index (THI) recorded in the control, moderate heat stress (MHS), severe heat stress (SHS), and relief group under severe heat stress (RHS) during the experimental period. THI < 72 = no stress, 72 to 79 = mild heat stress, 79 to 88 = moderate heat stress, >88 to serve heat stress.; Figure S2: Changes of the heat rate (A), respiratory rate (B), skin temperature (C), and rectal temperature (D) of lamb at 9:00, 13:00, 15:00, and 17:00 on the 4th day of test in each group; Figure S3: Summary of rarefaction results based on amplicon sequence variants ASVs for each sample; Figure S4: Principal coordinate analysis (PCoA) profile of microbial diversity using the Bray–Curtis dissimilarity metric; Table S1: Experimental diet composition and nutrient level (airdry basis); Table S2: Comparison of average daily feed intake (ADFI) and average daily weight gain (ADG) among the C, MHS, SHS and SHS groups; Table S3: Comparison of heart rate (HR), respiratory rate (RR), skin temperature (ST), and rectal temperature (RT) on the 3th, 4th and 5th day of the test at 15:00 in the MHS and SHS groups; Table S4: Summary of reads of bacteria from the ruminal digesta sample of lambs in the C, MHS, SHS and RHS groups; Table S5: Alpha diversity of bacterial community among each group; Table S6: The composition of predominant bacterial phyla in each group (the average relative abundance ≥1%among each group).
