# Supplementary material for: Folic Acid and Taurine Alleviate the Impairment of Redox Status, Immunity, Rumen Microbial Composition and Fermentation of Lambs under Heat Stress
Source: Animals (Basel). 2024 Mar 25;14(7):998. doi: 10.3390/ani14070998 (PMC11010938; doi:10.3390/ani14070998)
Supplement: Supplementary file 1 [file animals-14-00998-s001.zip › animals-2887656-supplementary.pdf]

.

# Folic Acid and Taurine Alleviate the Impairment of Redox Status, Immunity, Rumen Microbial Composition and Fermentation of Lambs under Heat Stress

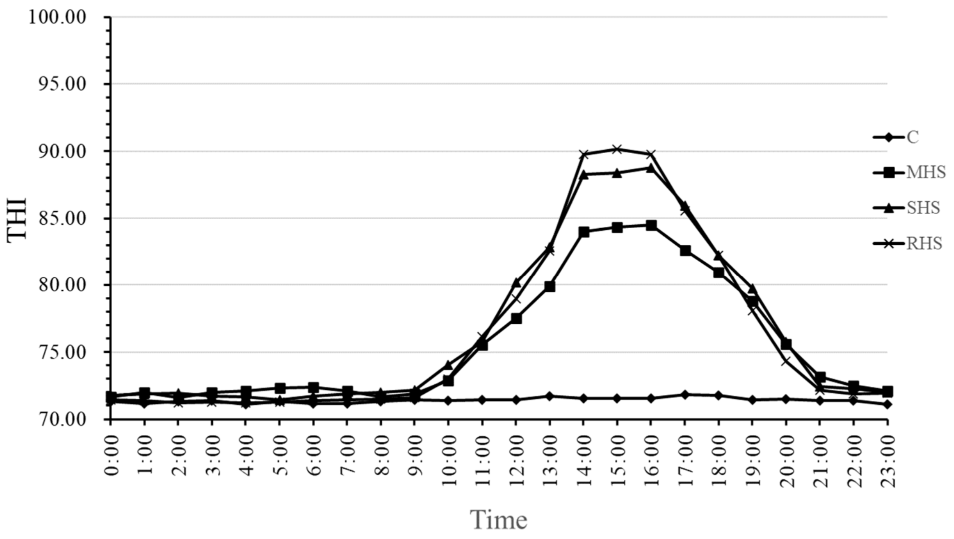


**Figure S1** Average daily temperature humidity index (THI) recorded in the control, moderate heat stress (MHS), serve heat stress (SHS), and relief group under serve heat stress (RHS) during the experimental period. THI < 72 = no stress, 72 to 79 = mild heat stress, 79 to 88 = moderate heat stress, >88 to serve heat stress.

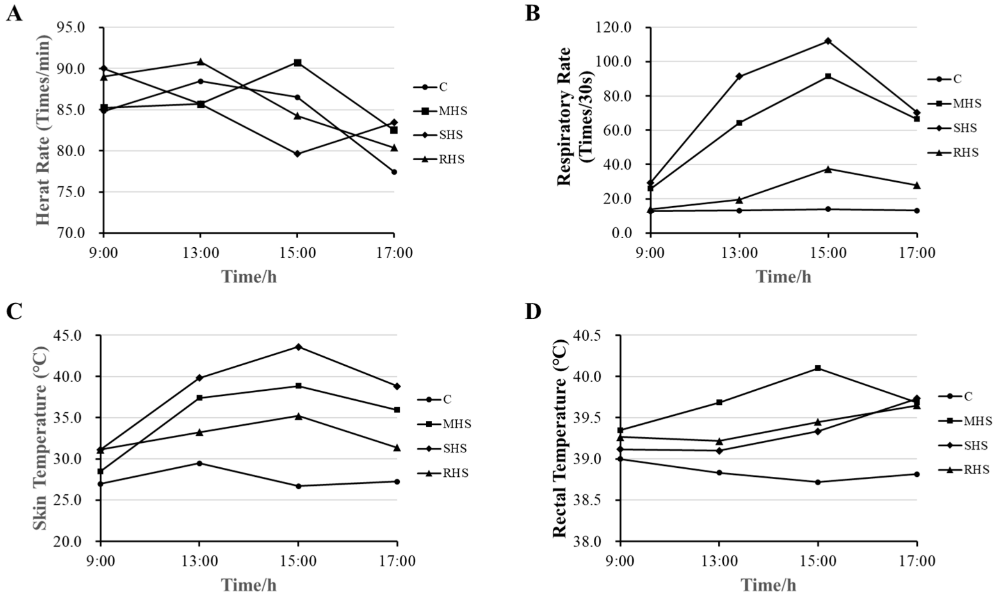


**Figure S2** Changes of the heat rate (A), respiratory rate(B), skin temperature(C), and rectal temperature (D) of lamb at 9:00, 13:00, 15:00, and 17:00 on the 4$^{th}$ day of test in each group.

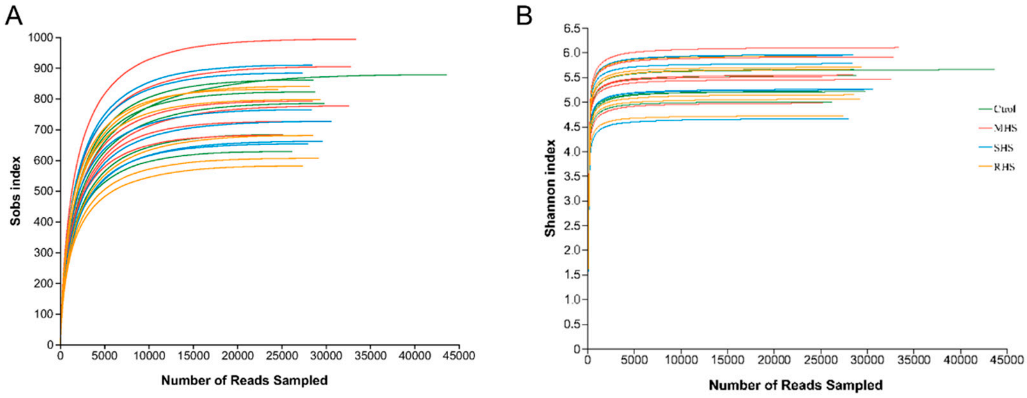


**Figure S3** Summary of rarefaction results based on amplicon sequence variants ASVs for each sample.

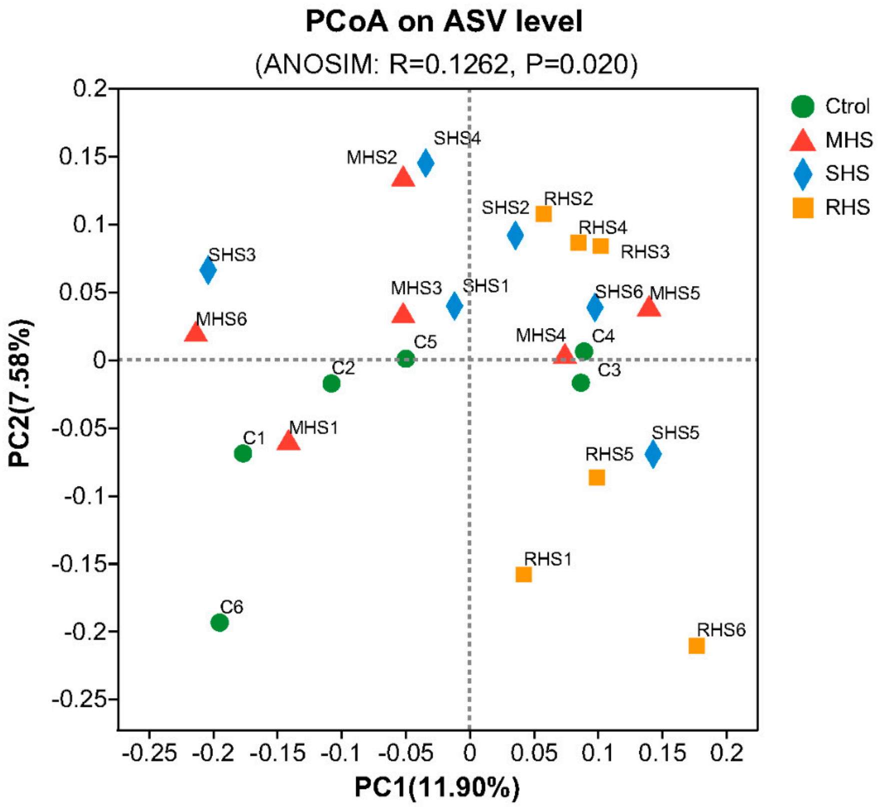


**Figure S4** Principal coordinate analysis (PCoA) profile of microbial diversity using the Bray-Curtis dissimilarity metric. The percentage of variation explained by PC1 and PC2 are indicated on the axis.

**Table S1** Experimental diet composition and nutrient level (airdry basis)

| Ingredients | Percent (%) | Nutrient levels [2)] | Contents |
|---|---|---|---|
| Corn straw | 33.50 | ME/ (MJ/Kg) | 9.17 |
| Alfalfa hay meal | 23.00 | CP /% | 12.11 |
| Corn | 28.00 | EE /% | 2.20 |
| Soybean meal | 9.00 | NDF /% | 41.53 |
| Wheat bran | 4.80 | ADF /% | 25.34 |
| Salt | 0.50 | Ca /% | 0.79 |
| Calcium hydrogen phosphate | 0.70 | P /% | 0.42 |
| Premix[1)] | 0.50 | | |
| Total | 100.00 | | |

1) The premix provided the following per kg of diets: Cu 5.84 mg, Fe 40 mg, Zn 25.6 mg, Mn 24.6 mg, Se 0.42 mg, I 0.4 mg, Co 0.24 mg, VA 2 400 IU, VD 160 IU, VE 240 IU.
2) ME was a calculated value, while the others were measured values.

**Table S2** Comparison of average daily feed intake (ADFI)and average daily weight gain (ADG) among in C, MHS, SHS and SHS group.

| Items | C | MHS | SHS | RHS | *P* |
|---|---|---|---|---|---|
| ADFI/(kg/d) | 1.14±0.01 | 1.05±0.10 | 1.11±0.02 | 1.20±0.02 | 0.276 |
| ADG/(g/d) | 225.40±9.45$^{c}$ | 103.33±68.18$^{a}$ | 119.00±19.75$^{a}$ | 161.40±20.34$^{b}$ | 0.028 |

ADFI, average daily feed intake; ADG, average daily weight gain.
Mean values with different superscripted lowercase letters within the same column differ significantly ($P < 0.05$).
C, means the control group; MHS, means the group of median heat stress; SHS, means the sever heat stress group; RHS, means the relief group under serve heat stress.

**Table S3** Comparison of heart rate (HR), respiratory rate (RR), skin temperature (ST), and rectal temperature (RT)among the 3th, 4th and 5th day of the test at 15:00 in MHS and SHS group.

| Day/d | HR /(time/min) | RR/ (times/30 s) | ST /℃ | RT /℃ |
|---|---|---|---|---|
| **MHS** | | | | |
| 3 | 89.17±3.95 | 78.11±5.45$^{a}$ | 38.17±0.47 | 39.87±0.35 |
| 4 | 90.72±3.88 | 91.33±8.47$^{b}$ | 38.87±0.27 | 40.10±0.39 |
| 5 | 92.83±4.69 | 74.78±5.06$^{a}$ | 37.27±1.21 | 39.68±0.27 |
| *P* | 0.826 | 0.008 | 0.360 | 0.695 |
| **SHS** | | | | |
| 3 | 79.61±3.59$^{a}$ | 108.17±10.47 | 43.58±0.61$^{ab}$ | 39.28±0.13 |
| 4 | 82.78±3.26$^{b}$ | 112.06±16.91 | 44.15±0.84$^{b}$ | 39.33±0.10 |
| 5 | 76.89±2.99$^{a}$ | 110.67±17.80 | 40.01±0.35$^{a}$ | 39.35±0.10 |
| *P* | 0.046 | 0.984 | 0.001 | 0.907 |

Mean values with different superscripted lowercase letters within the same colum differ

significantly ($P$ < 0.05).

**Table S4** Summary of reads of bacteria from the ruminal digesta sample of lambs in C, MHS, SHS and RHS group.

| **Samples** | **Prior to denoise** | **After denoise** | |
|---|---|---|---|
| | Reads | Reads | ASVs |
| **C** | | | |
| C1 | 46226 | 29726 | 784 |
| C2 | 45455 | 28560 | 861 |
| C3 | 46078 | 28663 | 822 |
| C4 | 46744 | 26059 | 628 |
| C5 | 69677 | 43619 | 878 |
| C6 | 38104 | 25120 | 683 |
| **MHS** | | | |
| MHS1 | 52388 | 32488 | 776 |
| MHS2 | 54979 | 33328 | 993 |
| MHS3 | 46256 | 28493 | 792 |
| MHS4 | 42672 | 25086 | 681 |
| MHS5 | 40093 | 25144 | 725 |
| MHS6 | 54031 | 32648 | 904 |
| **SHS** | | | |
| SHS1 | 45207 | 28303 | 764 |
| SHS2 | 50145 | 30478 | 726 |
| SHS3 | 48482 | 29436 | 661 |
| SHS4 | 51181 | 28454 | 909 |
| SHS5 | 46056 | 27979 | 653 |
| SHS6 | 44945 | 27257 | 883 |
| **RHS** | | | |
| RHS1 | 51844 | 29191 | 606 |
| RHS2 | 49914 | 29385 | 797 |
| RHS3 | 42669 | 28179 | 840 |
| RHS4 | 43650 | 24569 | 830 |
| RHS5 | 45018 | 28518 | 680 |
| RHS6 | 43981 | 27385 | 581 |

**Table S5** Alpha diversity of bacterial community among each group.

| Samples | Sobs | Chao | Shannon | Coverage |
|---|---|---|---|---|
| **C** | | | | |
| C1 | 784 | 784 | 5.20 | 1 |
| C2 | 861 | 861 | 5.66 | 1 |
| C3 | 822 | 822 | 5.53 | 1 |
| C4 | 806 | 806 | 5.00 | 1 |
| C5 | 878 | 878 | 5.65 | 1 |
| C6 | 683 | 683 | 5.21 | 1 |
| **MHS** | | | | |
| MHS1 | 776 | 776 | 5.45 | 1 |
| MHS2 | 744 | 744 | 5.37 | 1 |
| MHS3 | 792 | 792 | 5.54 | 1 |
| MHS4 | 681 | 681 | 5.45 | 1 |
| MHS5 | 725 | 725 | 4.97 | 1 |
| MHS6 | 744 | 744 | 5.37 | 1 |
| **SHS** | | | | |
| SHS1 | 764 | 764 | 5.78 | 1 |
| SHS2 | 726 | 726 | 5.26 | 1 |
| SHS3 | 661 | 661 | 5.24 | 1 |
| SHS4 | 909 | 909 | 5.96 | 1 |
| SHS5 | 653 | 653 | 4.65 | 1 |
| SHS6 | 883 | 883 | 5.94 | 1 |
| **RHS** | | | | |
| RHS1 | 606 | 606 | 5.05 | 1 |
| RHS2 | 797 | 797 | 5.70 | 1 |
| RHS3 | 840 | 840 | 5.66 | 1 |
| RHS4 | 701 | 701 | 5.23 | 1 |
| RHS5 | 680 | 680 | 5.14 | 1 |
| RHS6 | 701 | 701 | 5.23 | 1 |

**Table S6** The composition of predominant bacterial phyla in each group (the average relative abundance ≥1% among each group). (%)

| Taxa | C | MHS | SHS | RHS |
|---|---|---|---|---|
| p_Bacteroidota | 56.43 | 51.92 | 51.45 | 53.67 |
| p_Firmicutes | 35.20 | 43.98 | 44.64 | 41.67 |
| p_Proteobacteria | 4.07 | 0.21 | 0.32 | 0.60 |
| p_Spirochaetota | 1.07 | 1.52 | 1.04 | 1.27 |
| p_Actinobacteriota | 0.88 | 0.61 | 0.76 | 1.69 |
| others | 2.33 | 1.80 | 1.10 | 1.80 |
